# Chicago Medical Society

**Published:** 1871-01

**Authors:** 


### PROCEEDINGS OF THE CHICAGO MEDICAL SOCIETY.

CHICAGO, November 7, 1870.

*Dr. E. L. Holmes* reported a case of disease of the eye, in which, at first, the lens, by some wholly unaccountable means, passed out of its capsule into the anterior chamber. It afterward, somehow, replaced itself; was found within its capsule. Later—two years ago—the lens fell forward again, and became attached to the iris, probably by inflammatory adhesion; there it underwent calcarious degeneration, so that at the time of the recent treatment, it was perfectly opaque. There had been, during the two years, constant pain, of a most excruciating character, which, no sort and no amount of anodynes in the hands of attending physicians have been able to control, and hardly assuage. The Cataract was extracted, and a portion of the iris removed likewise, and the patient made a rapid recovery, and the neuralgic pains entirely ceased.

Dr. H. reported another case very similar to the above. The lens became detached from its capsule and floated in the aqueous humor, passing in and out of its capsule repeatedly, but finally underwent calcarious degeneration, and remained then in the anterior chamber. It gave rise to the same agonizing pain. On extraction the pain ceased and the patient recovered.

*Dr. J. G. Fredigke* remarked that patients not unfrequently have cataract in one eye, which entirely disables it for purposes of sight, yet, owing to its insiduous and slow growth, they are not aware of the fact until told of it or made to understand it by being asked to close the well eye and see with the other. He had seen a case of this sort.

*Dr. Holmes* said he had repeatedly seen cases of this kind. The patients are aware that something is the matter with their sight, but from always opening and closing both eyes at the same time, always closing both—indeed, when some irritating body suddenly strikes one or when rubbing or wiping one, they never become aware that one is wholly blind until informed of it. One patient of his had nearly fainted when made aware of such a condition.

*Dr. H. W. Boyd* said strabismic patients often lose the sight of the unused eye without being aware of the fact, and are only made conscious of it when, being operated upon, they endeavor to see with the affected eye.

*Dr. Fredigke* reported a case of fracture of both bones of the forearm at the centre—the ulna being fractured in two places, an inch apart, in a child five or six years old.

The usual retaining apparatus was applied, and union took place in twelve to fourteen days, when the splints were removed. During this time officious parents, contrary to the doctor's directions, themselves loosened the dressings, to relieve the child from suffering; the result of this was that the ulna was slightly bent, and remained so when it solidified. This caused the parties to be dissatisfied with the treatment.

*Dr. N. S. Davis* urged that surgeons should be more explicit in their directions not to have their dressings of fractures interfered with. Patients should be made to understand that any failure to carry out the surgeon's directions entirely released him from any responsibility in the case; and surgeons should, for self-protection, have more professional friends witness their cases after they have been tampered with by patients or nurses.

The President said that he did not believe perfect bony union could take place in a fractured bone of a child, even, in fourteen days; the case reported as such must have been one of firm cartialginous union, for bony union never takes place earlier than three weeks. He had himself charged a patient with fracture of the tibea near the ankle, which had been in splints 30 days, on putting on a starch bandage, that he must, when sitting down, with his limb at rest, not to allow the heel to hang over the chair, lest the weight of the foot should cause bending of the bone at the point of injury. The patient disregarded his injunction, and the leg became crooked.

*Dr. Davis* then related a case of supposed heart-clot and embolism. The patient was a Swede, a young man strong and healthy, usually; he was attacked with pneumonia, which involved almost, or quite, one entire lung. All the physical signs, both on percussion and auscultation, indicated this condition, as well as all the symptoms; and the prognosis was very unfavorable.

But the patient, for several days, made steady improvement, when, all at once, both arms began to be tumefied below the centre of the biceps muscle. Very soon they were both enormously swollen, and œdematous, and there was no pulse perceptible in the right wrist.

A feeble pulse could be felt in the left arm, and pulsation of the axillary arteries was palpable.

The heart indicated great labor, and strong effort on the part of that organ to force the blood through the system. He complained once or twice of difficulty in seeing. In two days, the lower limbs became swollen in the same way as the arms; this, however, occurred only below the knees.

For five days the patient improved; the tumifaction in the arms subsided, yet in the right there did not appear any pulse at the wrist, while in the left this was perceptible all the time. Very soon the legs began to show signs of improvement. The patient was improving rapidly in other respects, and was able to converse freely.

In three or four hours from the last visit, the patient was found by his nurse, after a few moments absence from the room, with the face perfectly turgid with blood, and he gasping for breath. In this state he died in a few minutes.

No *post mortem* examination could be made.

He reported another case of embolism, in a young girl, who was recovering from typhoid fever. She had manifested during the fever symptoms of weakness and inefficiency of heart's action, and had been taking mineral acid and strychnia.

When raised up, she gave evidence of syncope, yet convalesced steadily until able to set up, when she complained of pain in the right gluteal region, rather over the gluteus maximus, and, almost coincident with this, her leg began to swell, and became, ultimately, much tumified and œdematous. This remained some days, when it began to subside, and was, at this time, slowly disappearing.

*November* 21, 1870.

President, Dr. T. D. Fitch in the Chair.

*Dr. Reid* reported a case of spurious pregnancy in a woman of 32 years. The patient had borne two children, the youngest nine years before, and was pleased at the idea of being pregnant. Her menstruation did not wholly cease, but decreased very much. The fact that women do sometimes menstruate when pregnant was explained to her. Her abdomen enlarged, and after about four months she felt, as she supposed, the movements of the child. Her general health was good, bowels regular, and mind tranquil. When her nine months arrived, she had no symptoms of approaching confinement. At ten months, Dr. R. was sent for, to determine the reason for the delay. He found her enlarged to the size usual at term, but, on examination, the uterus was found not enlarged, and he stated to the woman that she was not pregnant.

This she would not believe, because she had felt, for months, distinctly, "the motions of the child," which were exactly like those experienced during former pregnancies.

There could be found no tumor of any circumscribed or well-defined character, in the abdomen; there was no fluctuation; but a distinct tympanitic sound could be heard on percussing over parts of the abdomen. There could be learned no general symptom that could indicate the existence of a tumor of any sort.

After a short time, she began slowly to decrease in size, and in a few more months, was little larger than normal. What was the cause of the enlargement?

*Dr. Marguerat* had seen a case somewhat resembling this. A very fleshy woman stopped menstruating, and began to enlarge. She was told by one or two physicians, whom she consulted, that she was not; yet she was herself certain of it, having felt, as she supposed, the motions of the child.

The Doctor was called suddenly to attend her, labor having, as it was supposed, begun. A large amount of blood was being discharged per vaginum, and nothing else, and there was no pregnancy, which divulged a mental element, that lent some explanation to the cause.

*The President* related a case, not unlike the last, of a woman 35 years old, who was childless, and exceedingly anxious to become a mother. Her menstruation ceased, her abdomen enlarged and pregnancy was supposed to exist. After the usual time, she was taken with pain and discharges of blood. A homœpathic physician was called; he made an examination, and told the friends that labor was progressing, and that everything was "right." He remained with her all night; labor did not progress rapidly, and he left, and called later in the day, to find little progress had been made.

The result of the case was, that the bleeding continued for a number of days, the woman's abdomen decreased, and she has never been delivered of a child.

*Dr. Reid* reported a case of supposed ovarian tumor in a young woman. The enlargement on palpation and percussion manifested the usual signs of a unilocular tumor; it seemed to have a distinct attachment to the side of the uterus or the broad ligament. Yet, after a few months, it began to subside, and was now almost entirely gone.

*Dr. Paoli* had known of a case, where, after delivery, a tumor was found attached to the uterus, of the size of a fœtal head, which disappeared gradually without treatment.

*Dr. Marguerat* detailed a case of the same character as that of Dr. Paoli's, only in this the tumor developed after delivery, not being detected at the confinement. It was attached to the left broad ligament. Six weeks after delivery, was as large as a child's head, but gave no evidence of fluid when percussed.

Under the use of friction and the internal administration of mercury, the growth disappeared.

*Dr. Ferine* had known of a case in the Cook County Hospital, in which an ovarian tumor was supposed to exist in a woman delivered only a few months before. A consultation was called, to determine the time of operating, when it was thought best to give the patient a cathartic. A large quantity of fecal matter, which had distended the colon, was discharged, and the tumor was gone. —*Chicago Medical Examiner.*

